# STAT1 Pathway Mediates Amplification of Metastatic Potential and Resistance to Therapy

**DOI:** 10.1371/journal.pone.0005821

**Published:** 2009-06-08

**Authors:** Nikolai N. Khodarev, Paul Roach, Sean P. Pitroda, Daniel W. Golden, Mihir Bhayani, Michael Y. Shao, Thomas E. Darga, Mara G. Beveridge, Ravi F. Sood, Harold G. Sutton, Michael A. Beckett, Helena J. Mauceri, Mitchell C. Posner, Ralph R. Weichselbaum

**Affiliations:** 1 Department of Radiation and Cellular Oncology, University of Chicago, Chicago, Illinois, United States of America; 2 Department of Surgery, University of Chicago, Chicago, Illinois, United States of America; Roswell Park Cancer Institute, United States of America

## Abstract

**Background:**

Traditionally IFN/STAT1 signaling is connected with an anti-viral response and pro-apoptotic tumor-suppressor functions. Emerging functions of a constitutively activated IFN/STAT1 pathway suggest an association with an aggressive tumor phenotype. We hypothesized that tumor clones that constitutively overexpress this pathway are preferentially selected by the host microenvironment due to a resistance to STAT1-dependent cytotoxicity and demonstrate increased metastatic ability combined with increased resistance to genotoxic stress.

**Methodology/Principal Findings:**

Here we report that clones of B16F1 tumors grown in the lungs of syngeneic C57BL/6 mice demonstrate variable transcriptional levels of IFN/STAT1 pathway expression. Tumor cells that constitutively overexpress the IFN/STAT1 pathway (*STAT1^H^* genotype) are selected by the lung microenvironment. *STAT1^H^* tumor cells also demonstrate resistance to IFN-gamma (IFNγ), ionizing radiation (IR), and doxorubicin relative to parental B16F1 and low expressors of the IFN/STAT1 pathway (*STAT1^L^* genotype). Stable knockdown of *STAT1* reversed the aggressive phenotype and decreased both lung colonization and resistance to genotoxic stress.

**Conclusions:**

Our results identify a pathway activated by tumor-stromal interactions thereby selecting for pro-metastatic and therapy-resistant tumor clones. New therapies targeted against the IFN/STAT1 signaling pathway may provide an effective strategy to treat or sensitize aggressive tumor clones to conventional cancer therapies and potentially prevent distant organ colonization.

## Introduction

Metastasis is the primary cause of death in most cancer patients. The treatment of patients with metastatic disease in adult solid tumors remains largely palliative. The ineffectiveness of standard cancer therapies against metastatic disease is reflected by the marked disparity in survival from the time of diagnosis for localized versus distant disease [1]. This disparity is hypothesized to be due to a greater disease burden in patients with metastatic disease and the intrinsic resistance of metastatic disease to most cancer therapies. Metastasis resistance to conventional therapy is believed to be due to the multiple genetically unstable cell populations found within tumors [2]. Though there are many investigations into the mechanisms of metastasis, little is known regarding the mechanisms of metastatic progression that contribute to therapy resistance.

One key selection pressure shaping tumor cell evolution is the tumor microenvironment, which includes tumor cells, host stromal cells, extracellular matrix, and cells of the immune system. One component of the interaction between the tumor and the microenvironment is mediated by the immune system via “immunoediting” [3]. Immunoediting is proposed to be the process by which the immune system drives tumor cell selection towards an immune-resistant phenotype [3], [4], including resistance to the multiple host-secreted cytokines.

A key cytokine in tumor-suppressive networks is IFN-gamma (IFNγ) [5]. IFNγ mediates its effect on cells by interacting with type II IFN receptors (IFNGR1 and IFNGR2). Upon receptor binding, IFNγ activates the JAK/STAT1 dependent signaling pathway.

STAT1, the first described member of the STAT transcription factor family [6], is the master transcription factor for IFN-related intracellular signaling and therefore tumor suppression related to IFNs. STAT1 is phosphorylated by JAK1/2 kinases at the Tyr701 position and then translocates to the nucleus where it binds to GAS (IFNγ-activated sequence) promoter elements, thereby activating several hundred genes. These interferon-stimulated genes comprise the IFN/STAT1 signaling pathway [7], [8]. Therefore, the IFN/STAT1 pathway represents a signaling pathway that mediates crosstalk between the host microenvironmental components and the tumor cells.

Genes activated by STAT1 determine many of the functions related to the IFN/STAT1 pathway. Previous studies demonstrated that STAT1 controls anti-tumorigenic effects in part by up-regulation of caspases 1, 2, 3, 7, and 8 [9]–[12], cyclin-dependent kinase inhibitor 1A (CDKN1A) [13], the IFN-regulatory Factor 1 (IRF1)/p53 pathway [14], and down-regulation of the BCL2 (B-cell CLL/Lymphoma 2) family [15]. STAT1 also is involved in anti-angiogenic mechanisms, partially through the induction of IFNγ-induced protein 10 (IP10 or CXCL10), thereby suppressing tumorigenesis [16].

In contrast, emerging data reveal that in certain cellular contexts the IFN/STAT1 pathway may mediate tumor cell growth. Previously, we demonstrated that a radioresistant tumor (Nu61), selected against fractionated ionizing radiation (IR) from a radiosensitive parental tumor SCC61, constitutively overexpresses the IFN/STAT1 pathway [17]. The nu61 radioresistant tumor and corresponding cell lines were also found to be resistant to IFN-mediated cytotoxicity [18]. A subsequent study demonstrated that selection of SCC61 against IFN-alpha (IFNα) and IFNγ produced a similar IR- and IFN-resistant phenotype overexpressing the IFN/STAT1 pathway [18]. Thus, we concluded that resistance to IR and IFNs is associated with constitutive overexpression of the IFN/STAT1 pathway. Consistent with our observations are recent reports indicating that constitutive overexpression of STAT1 and STAT1-dependent genes is associated with protection of tumor cells from genotoxic stress following treatment with fludarabine [19], doxorubicin (Dox) [20], cisplatin [21], and the combination of IR and Dox [22], [23]. Overexpression of the IFN/STAT1 pathway is also associated with poor prognosis in different types of cancer and may have predictive value in breast cancer patients selected for the adjuvant chemotherapy [24], [25].

Based on these findings, we hypothesized that *in vivo* selection of developing tumor clones would be associated with the ability of tumor cells to overexpress the IFN/STAT1 pathway while resisting IFNγ-mediated cytotoxicity. To test this hypothesis we used the syngeneic B16Fl mouse melanoma lung colonization model as a surrogate for metastatic propensity. This model was developed and described in detail by Fidler and colleagues [26]–[28] (see [2] for review). We investigated whether differences in phenotype between parental B16F1 and its derivatives passed through the lungs are related to IFNγ-resistance, IFN/STAT1 pathway overexpression, and resistance to cytotoxic therapy including IR and chemotherapy.

Here we report that tumor cells that constitutively overexpress the IFN/STAT1 pathway, and are thus resistant to the cytotoxic effects of this pathway, are preferentially selected for colonization by the lung microenvironment. Clones overexpressing this pathway produce a greater number of lung colonies compared to clones that do not constitutively overexpress the IFN/STAT1 pathway. We demonstrate the pre-existence of clones in the B16F1 tumor cell population that overexpress the IFN/STAT1 pathway. These clones are also resistant to IR and Dox. Suppression of *STAT1* by stable knockdown using small hairpin RNA (shRNA) leads to a reversion of the phenotype, decreased ability to form lung colonies and decreased resistance to genotoxic stress. These data provide a mechanistic explanation for the STAT1-dependence of pro-metastatic and therapy-resistant tumor clones. We propose that overexpression of the IFN/STAT1 pathway contributes to therapy resistance and metastatic propensity leading to the poor response to traditional cancer therapy observed in patients with metastatic disease.

## Results

### Efficiency of lung colonization is accompanied by development of resistance to IFNγ

**Figure 1:**
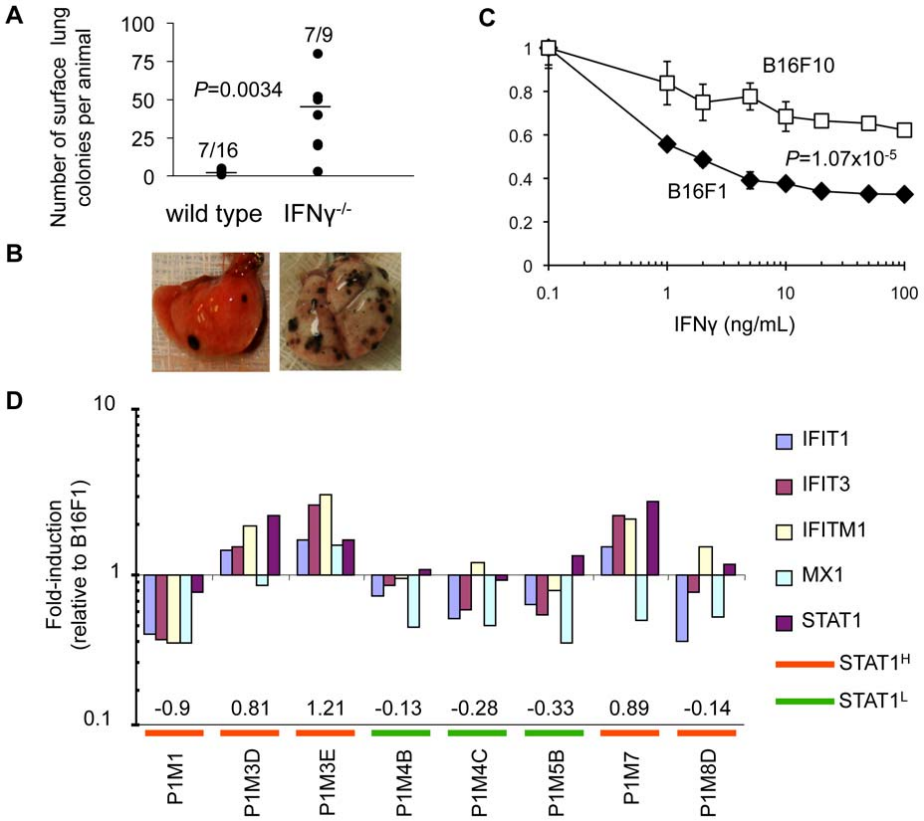
IFN/STAT1 signaling differs between tumor cell lines and is required for suppression of lung colonization. (A) Number of mice developing lung colonies and total number of lung colonies per mouse in C57BL/6 wild type and IFNγ^−/−^ mice after intravenous injection of B16F1 tumor cells (horizontal line represents mean). (B) Images of wild type and IFNγ^−/−^ explanted lungs demonstrate marked difference in number of lung colonies. (C) Differential response to IFNγ *in vitro* between B16F1 (parental) and B16F10 (passaged, more aggressive) cell lines (mean±SD). (D) Variable fold-induction of IFN/STAT1 marker genes for eight cultured cell lines from P1 passage B16F1 lung colonies in C57BL/6 mice (numbers represent expression scores).

We examined lung colonization following injection of B16F1 melanoma cells in IFNγ^−/−^ mice compared with C57BL/6 controls. After injection of 5×10^4^ B16F1 cells, 7/9 IFNγ^−/−^ mice developed lung colonies (mean number of lung colonies per mouse = 37.6±25.6). C57BL/6 mice developed significantly fewer lung colonies (7/16, number of lung colonies per mouse = 2.1±1.2; Student's two-tailed t-test; p = 0.0034; **Figure 1A-B**). These observations are consistent with prior reports demonstrating that IFNγ^−/−^ mice develop a greater number of lung colonies than wild type mice [5], [29], [30]. Passage of B16F1 cells through the lungs led to the formation of the highly metastatic daughter tumor B16F10 [26]. We suggested that such passages should lead to an increase in resistance to IFNγ and we compared the sensitivity of B16F1 and B16F10 cells to IFNγ with a MTS (3-(4,5-dimethylthiazol-2-yl)-5-(3-carboxymethoxyphenyl)-2-(4-sulfophenyl)-2H-tetrazolium) assay. We found that B16F10 cells were significantly more resistant to IFNγ that B16F1 cells (Student's two-tailed t-test; p = 1.07×10^−5^; **Figure 1C**). Taken together these results confirm the importance of IFNγ in suppressing B16F1 lung colonization and suggest that lung colonization is associated with resistance to IFNγ.

### Quantification of IFN/STAT1 pathway expression and definition of *STAT1^H^* and *STAT1^L^* genotype

The IFN/STAT1 pathway mediates transduction of IFN signals into the nuclei of target cells. This transduction involves activation of Type I and Type II IFN receptors, receptor-associated JAK/TYK (tyrosine kinase) kinases and eventually activation and nuclear translocation of STAT1. IFN-mediated STAT1 transcriptional induction results in the transcription of several hundred genes designated the Interferon-Stimulated Genes (ISG; see [8], [31], [32] for reviews). To define a cut-off expression level to delineate high and low expressors of the IFN/STAT1 pathway we developed a method for rapid and efficient screening of cell lines and tumor clones. Prior studies demonstrate that activation of the IFN/STAT1 pathway can be measured by PCR-based quantification of gene expression using marker ISGs [33]–[36].

We selected five genes – *STAT1, IFIT1* (interferon-induced protein with tetratricopeptide repeats 1), *IFIT3* (interferon-induced protein with tetratricopeptide repeats 3), *IFITM1* (interferon induced transmembrane protein 1), and *MX1* (Myxovirus resistance protein 1) – to comprise our IFN/STAT1 pathway marker gene set based on following criteria. First, the literature confirms that these five genes are PCR–detectable markers of the IFN/STAT1 pathway. Specifically data have been published for *STAT1* [34], *MX1* [34], [35], *IFITM1* [37], *IFIT1*, and *IFIT3* [36], [37]. Secondly, we calculated the correlation between our DNA array data from our originally described interferon-related gene signature [17] with QRT-PCR (quantitative real time PCR) amplification data of 19 different ISGs included in this signature. Our results demonstrate that the five marker genes selected for the current study are located within the 95% confidence interval (Pearson's R = 0.803, p<0.001, **Figure S1**). Third, we tested these five IFN/STAT1 pathway marker genes in multiple cell lines and xenografts. We found that these five genes were amplified in response to IFNs and/or IR in 3 of 3 cell lines and 7 of 8 xenografts tested [18]. Finally, we recently reported a statistically derived seven-gene TSP (top scoring pair) classifier related to ISGs that is associated with poor prognosis and chemo- and radio-resistance in tumors [25]. Four out of seven TSP genes (*STAT1, IFIT1, IFIT3* and *MX1*) overlap with our five-gene IFN/STAT1-pathway marker set (see above). Expression of each marker gene was normalized to B16F1 and the expression of all five genes was combined to generate our IFN/STAT1 expression score [33], [38]. This score was calculated as an average of the log_2_-transformed expression value for each of the five marker genes (see Methods for details) and was used to estimate the IFN/STAT1 pathway expression level in experimental cell lines and tumor clones.

To test whether the expression score, as defined above, discriminates IFN/STAT1 pathway high (*STAT^H^*) and low (*STAT^L^*) expressors relative to B16F1 baseline, we performed QRT-PCR to profile eight cell lines from the P1 passage (B16F1 passed through the lungs of wild type C57BL/6 mice; **Figure 1D** **and** **Figure S2**). Three of these cell lines (P1M3D, P1M3E, and P1M7) demonstrated an up-regulation of the five-gene marker set relative to B16F1 and had positive IFN/STAT1 pathway expression scores (0.81, 1.21 and 0.89; **Figure 1D**). These cell lines were defined as overexpressors of IFN/STAT1 pathway (*STAT1^H^*; **Figure 1D**). The other five cell lines (P1M1, P1M4B, P1M4C, P1M5B and P1M8D) did not change or exhibited a down-regulation of the five-gene marker set relative to B16F1. These cell lines showed negative IFN/STAT1 pathway expression scores ranging from −0.13 to −0.9 and were defined as low expressors (*STAT1^L^*; **Figure 1D**). The difference between scores for *STAT1^H^* and *STAT1^L^* cell lines was significant (Student's two-tailed t-test; p = 0.0007; **Figure 1D**). We set the threshold expression score separating *STAT1^H^* and *STAT1^L^* genotypes at 0.8, which approximates the lowest score of the three *STAT1^H^* P1 cell lines (P1M3D cell line, **Figure 1D**). Subsequently, cell lines were designated *STAT1^H^* if their IFN/STAT1 pathway expression score was≥0.8 and *STAT1^L^* if their IFN/STAT1 pathway expression score was <0.8.

### Constitutive overexpression of the IFN/STAT1 pathway is associated with B16 melanoma lung colonization

**Figure 2:**
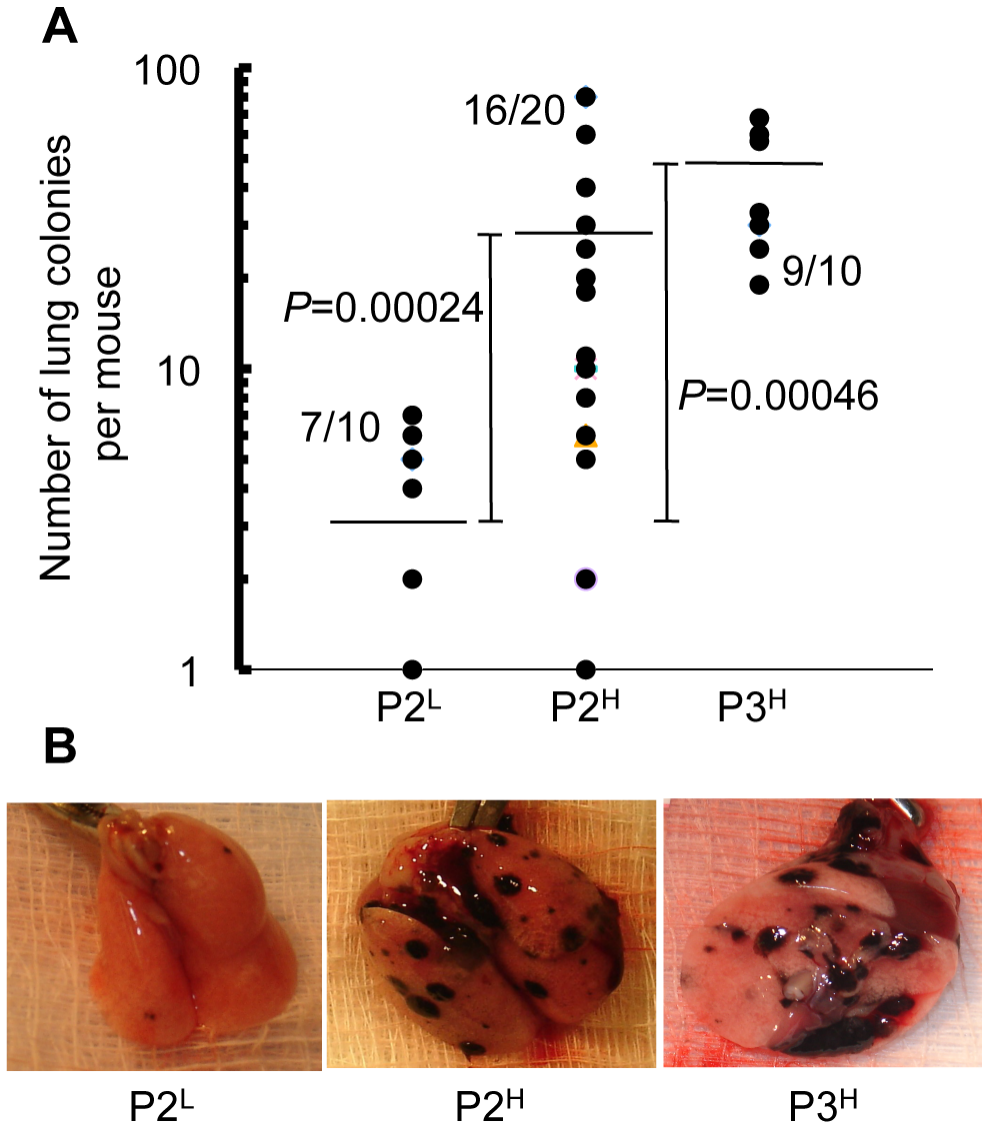
Passage of B16 cell lines based on IFN/STAT1 pathway expression level coincides with an increase in number of lung colonies. (A) Number of lung colonies per mouse after intravenous injection of 5×10^4^ tumor cells (horizontal line = mean number of lung colonies per mouse). (B) Representative images of lungs explanted from mice intravenously injected with 5×10^4^ P2^L^, P2^H^, or P3^H^ tumor cells, respectively.

We tested the hypothesis that tumor clones overexpressing the IFN/STAT1 pathway (*STAT1^H^*) exhibit an increase in lung colonization compared to cells that do not overexpress this pathway (*STAT1^L^*). Three P1 *STAT1^H^* clones (P1M3D, P1M3E, and P1M7) were pooled as were three P1 *STAT1^L^* clones (P1M4B, P1M4C, and P1M5B) and then injected into mice. In the P2 group of mice injected with the P1 *STAT1^L^* clones, 7 of 10 animals developed 2.8±2.5 lung colonies per animal which is equivalent to the number of lung colonies for parental B16F1 (2.1±1.2; 7/16 mice; Figure 2). By contrast, P2 mice injected with P1 *STAT1^H^* clones showed a marked increase in both the frequency of colony formation (16/20 mice; Fisher's exact test; p = 0.023) and the number of lung colonies per mouse (31.0±31.8; Student's two-tailed t-test; p = 2.4x10^−4^; **Figure 2**). These data indicate that the expression level of the IFN/STAT1 pathway affects the ability of B16F1 cells to colonize the lungs following IV injection.

### Propagation of the *STAT1^H^* genotype in tumor passages

**Figure 3:**
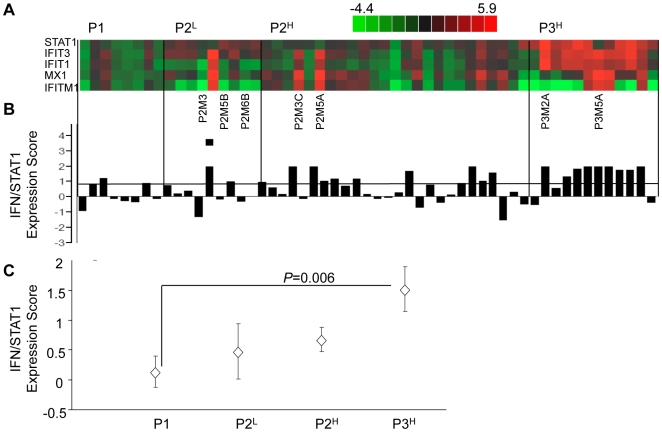
IFN/STAT1 pathway expression level and frequency of *STAT1^H^* clones increases with passage number. (A) Individual gene heat map and (B) STAT1 expression score of IFN/STAT1 pathway based on QRT-PCR expression levels for P1, P2^L^, P2^H^, and P3^H^ cell lines. (C) Mean IFN/STAT1 pathway expression score for P1, P2^L^, P2^H^, and P3^H^ cell lines (mean±SEM). See Table S1 for a description of cell lines and raw data.

The lung colonies produced by P1 *STAT1^H^* and P1 *STAT1^L^* cell lines were then used to generate a P2 panel of cell lines (**Figure 2** **and** **Figure S2**). Nine cell lines were isolated from P2 *STAT1^L^* colonies and 25 cell lines from P2 *STAT1^H^* colonies (**Figure 3A**). The two cell lines from the P2^H^ panel with the highest *STAT1* expression score, P2M3C (expression score = 2.36) and P2M5A (expression score = 2.87; **Figure 3A**) were pooled and injected intravenously into mice. These two pooled cell lines produced lung colonies in 90% (9/10) of mice with 37.9±18.7 colonies per mouse (p = 0.0046 compared with P2^L^; **Figure 2**). Lung colonies derived from this third *in vivo* passage are indicated as P3^H^ and 14 cell lines were cultured from these lung colonies (**Figure 3A**). All cell lines derived from *in vivo* passages are summarized in **Table S1**.

The mean *STAT1* expression score shows a progressive increase from the P1 to the P3 passage with a significant difference between the P1 and P3 panels of cell lines (Student's two-tailed t-test; p = 0.006; Figure 3C). In the P1 cell lines, 3/8 (37.5%) of the cell lines exhibited a *STAT1* expression score ≥0.8, classifying them as *STAT1^H^*. Of the P2^L^ cell lines only 2/9 (22.2%) represented *STAT1^H^* genotype while in the P2^H^ cell lines 11/25 (44%) belonged to *STAT1^H^* genotype. In the P3 cell lines 9/13 (69.2%) exhibited the *STAT1^H^* genotype. Thus, an increase in the frequency of the *STAT1^H^* cell lines and an increase in the mean level of *STAT1* expression scores occurs during the progression from P1 to P3 (**Figure 3A-C**). These results indicate that B16F1 lung colony forming efficiency is significantly associated with expression levels of the IFN/STAT1 pathway.

### Tumor clones overexpressing IFN/STAT1 pathway pre-exist in B16F1 population

**Figure 4:**
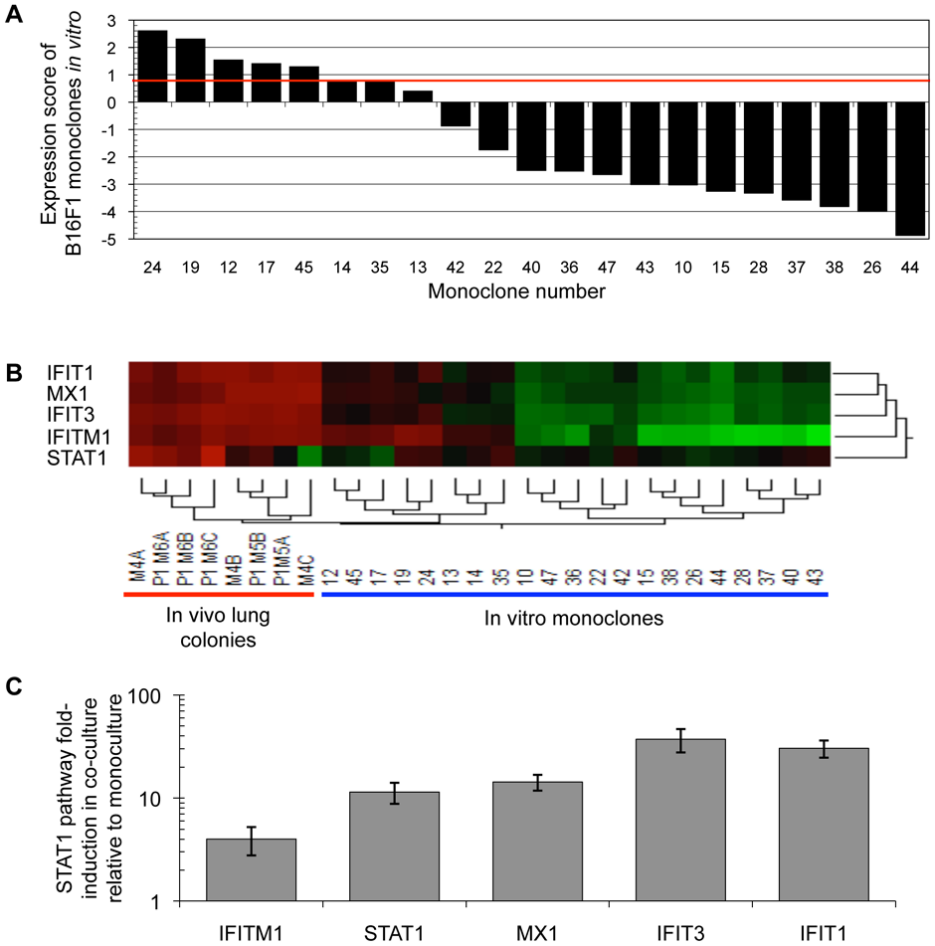
*STAT1^H^* variants pre-exist in the B16F1 population and the lung microenvironment stimulates the IFN/STAT1 pathway. (A) *STAT1^H^* variants pre-exist in a B16F1 population (red line represents expression score cutoff of 0.8 for *STAT1^H^* or *STAT1^L^*). (B) *In vivo* lung colonies have a markedly increased IFN/STAT1 pathway expression level compared with *in vitro* monoclones. (C) Induction of IFN/STAT1 pathway after *in vitro* co-culture of B16F1 with explanted lung tissue demonstrates a significant increase relative to B16F1 monocultures (p = 0.0174; mean±SD).

It was previously demonstrated that B16F1 cells have variable abilities to colonize the lungs due to preexisting heterogeneity of the tumor cell population [39]. It was suggested that the observed variability in B16F1 lung colonization proficiency might be connected with pathways that confer an increased capacity to colonize the lungs of C57BL/6 mice. In the current report we show that the ability to colonize the lungs is associated with constitutive overexpression of the IFN/STAT1 pathway (*STAT1^H^* genotype). We therefore hypothesized that the B16F1 cell population may contain pre-existent cells with a *STAT1^H^* genotype. To test this hypothesis we cloned B16F1 cells (see Methods) and investigated the expression levels of the IFN/STAT1 pathway in the progeny of monoclones. Five of 20 tested monoclones were *STAT1^H^*, and therefore overexpressed the IFN/STAT1 pathway (Student's two-tailed t-test; p = 5.33×10^−5^; **Figure 4A**).

We further compared *in vitro* monoclones to *in vivo* lung colonies with respect to expression of the IFN/STAT1 pathway. Eight lung colonies profiled *in vivo* demonstrated up-regulation of the IFN/STAT1 pathway from 60- to 120-fold relative to basal B16F1 *in vitro* depending on the marker genes (**Figure 4B**). On the contrary, *STAT1^H^* cell lines from the P1 panel (**Figure 1D**) and B16F1 monoclones (**Figure 4A**) exhibited up-regulation of the IFN/STAT1 pathway only 2 to 3-fold relative to baseline. These data indicate that the lung microenvironment uniformly up-regulates the IFN/STAT1 pathway in lung colonies.

**Figure 5:**
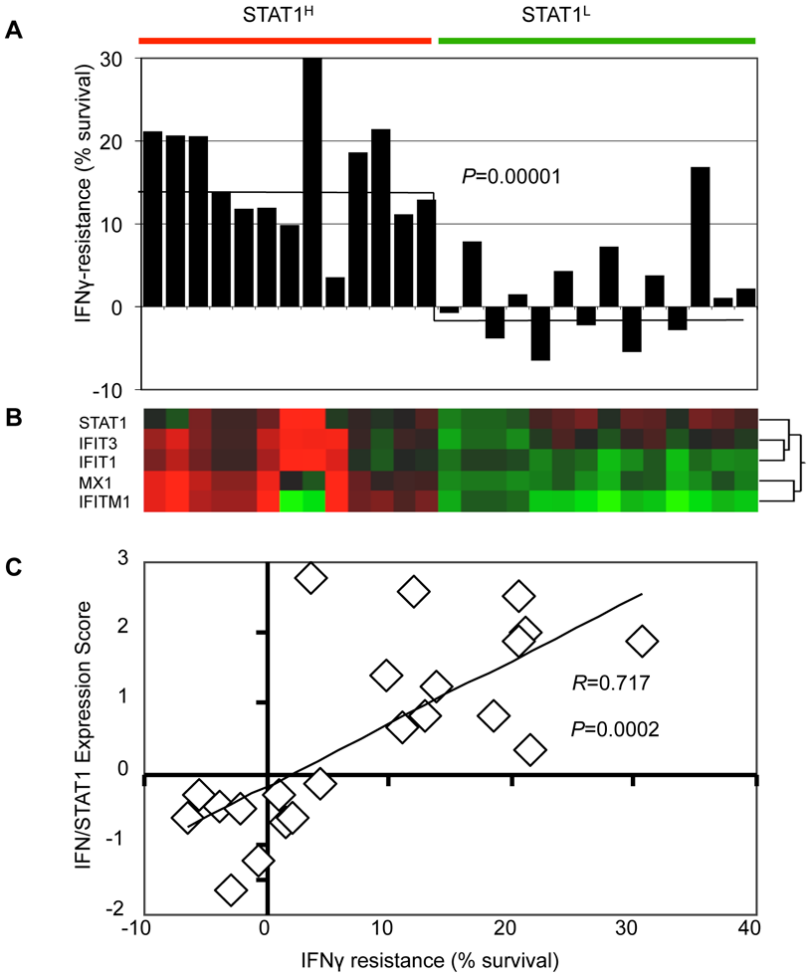
Resistance to IFNγ correlates with expression of IFN/STAT1 pathway. (A) Survival of STAT1^H^ and STAT1^L^ cell lines relative to control B16F1 after treatment with 10 ng/ml of IFNγ (horizontal line represents mean value). (B) Gene expression heat map of *STAT1^H^* and *STAT1^L^* cell lines used for IFNγ resistance experiment. (C) Correlation between IFN/STAT1 pathway expression level and resistance to IFNγ (see Table S2).

To confirm that the observed pathway up-regulation was directly related to factors from the lung microenvironment, B16F1 was co-cultured with explanted lung tissue (see Methods and [40], [41]). B16F1 co-culture with explanted lung tissue led to a marked (4 to 37 fold) up-regulation of the IFN/STAT1 pathway compared with B16F1/B16F1 mono-culture (Student's two-tailed t-test; p  = 0.0174; Figure 4C). It is noteworthy that IFN/STAT1 pathway up-regulation in co-culture is similar to the difference in expression between B16F1 monoclones *in vitro* and P1 lung colonies *in vivo* (**Figure 4B**). Two important conclusions were made from these experiments. First, the population of B16F1 cells is heterogeneous with respect to the IFN/STAT1 pathway expression and contains pre-existing *STAT1^H^* variants prior to *in vivo* passage. According to our observations, these cells are likely resistant to IFNγ cytotoxicity ([17], [18] and **Figure 5**). The second conclusion is that the lung microenvironment significantly up-regulates the IFN/STAT1 pathway in tumor clones, which may create a selection pressure and provide a survival advantage to the preexisting *STAT1^H^* clones that are inherently resistant to this cytotoxic microenvironment.

### *STAT1^H^* tumor clones are resistant to IFNγ

To test the hypothesis that overexpression of the IFN/STAT1 pathway in B16F1 cells correlates with IFNγ resistance (see above), *STAT1^H^* and *STAT1^L^* cell lines from all three passages were challenged with 10 ng/mL of IFNγ and viability assayed using MTS (see Methods). The mean viability of *STAT1^H^* clones was 16.02±6.7%, while that of *STAT1^L^* clones was 1.66±6.21% (Student's two-tailed t-test; p = 0.00001; **Figure 5A** **;** **Table S2**). Furthermore, IFNγ-resistant cell lines co-clustered with *STAT1^H^* cell lines (**Figure 5B**). Lastly, there was a strong correlation between *STAT1* expression score and resistance to IFNγ (Pearson's R = 0.717; p = 0.0002; **Figure 5C**). These results demonstrate that *STAT1^H^* tumor clones are more resistant to IFNγ compared with *STAT1^L^* tumor clones. Taken together with the data presented above, these findings indicate that overexpression of the IFN/STAT1 pathway is associated with resistance to IFNγ.

### Resistance to IFNγ in *STAT1^H^* tumor clones is also associated with resistance to ionizing radiation

We previously reported that selection of the radioresistant tumor nu61 by *in vivo* fractionated IR was accompanied by overexpression of the IFN/STAT1 pathway and combined resistance to IR, IFNα, and IFNγ [17], [18]. We also showed that selection of the sensitive parental tumor SCC61 against IFNα or IFNγ led to constitutive overexpression of the IFN/STAT1 pathway and development of resistance to IR [18]. We therefore hypothesized that *STAT1^H^* B16 clones having an increased propensity to colonize the lungs and demonstrating resistance to IFNγ, would also grow more rapidly and be resistant to IR.

**Figure 6 pone-0005821-g006:**
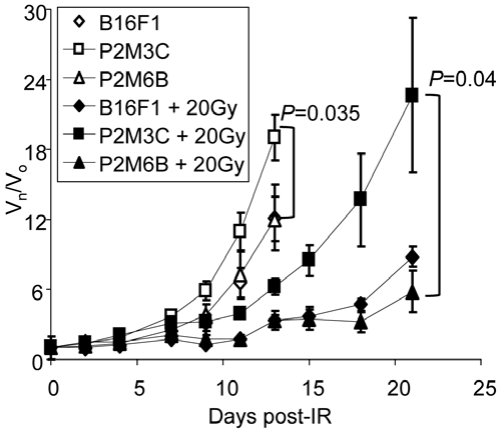
*STAT1^H^* tumors are resistant to IR. STAT1^H^ (P2M3C) tumors grow faster and are resistant to IR compared with STAT1^L^ (P2M6B) and B16F1 parental tumors. (V_n_/V_o_ = relative change in volume, day 0 = IR (20Gy), mean±SEM)

To test this hypothesis, we compared two cell lines derived from second generation lung colonies (P2) in which P2M3C was derived from pooled P1 *STAT1^H^* cell lines (P2^H^) and P2M6B was derived from pooled P1 *STAT1^L^* cell lines (P2^L^) (**Figure 3** **and** **Figure S2**). Also, P2M3C (P2^H^) was previously shown to be resistant to IFNγ, while P2M6B (P2^L^) was sensitive to IFNγ (**Figure 5** **and** **Table S2**). The parental B16F1 cell line was used as a control. Experiments were performed *in vivo* using hind limb injections of tumor cells into C57BL/6 mice as previously described [42] (see Methods). At day 13 after hind limb injection, the relative volume [43] of untreated P2M3C (*STAT1^H^*) tumors was 19.0±1.97 while that of P2M6B (*STAT1^L^*) was 12.0±1.92 (1.6-fold difference; Student's two-tailed t-test; p = 0.035). These data demonstrate that P2M3C (*STAT1^H^*) grew significantly faster compared to P2M6B (*STAT1^L^*) and B16F1 (**Figure 6**).

Tumors irradiated with 20 Gy demonstrated a similar trend. At day 21 after IR the relative tumor volume of irradiated P2M3C was 3.9 times larger (22.7±6.67) than the relative volume of P2M6B (5.8±1.78; Student's two-tailed t-test; p = 0.04; **Figure 6**). Thus, after a single dose of 20 Gy, the differences in relative volumes increased between tumors derived from *STAT1^H^* and *STAT1^L^* cell lines. Therefore, consistent with our previous observations [17], [18], tumors overexpressing the IFN/STAT1 pathway are resistant to both IR and IFNγ.

### *STAT1^H^* clones demonstrate resistance to Doxorubicin

**Figure 7:**
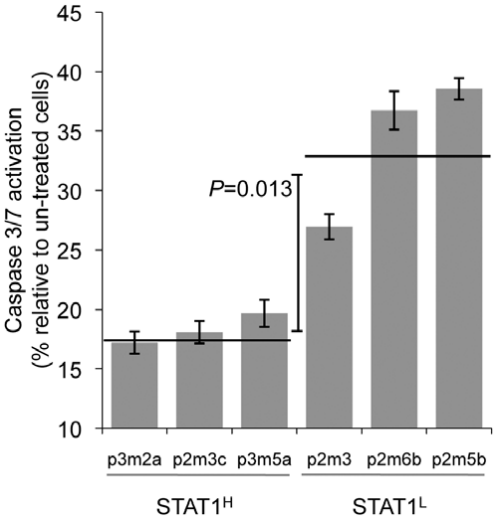
*STAT1^H^* cell lines are resistant to doxorubicin-induced apoptosis. Caspase 3/7 activation as a measure of apoptosis in cells exposed to doxorubicin (1 µM) for 24 hours (mean±SD, horizontal line represents mean for *STAT1^H^* and *STAT1^L^* groups). Data are presented as fold-induction relative to control (un-treated) cells.

We previously demonstrated that *STAT1^H^* tumors overexpressing the IFN/STAT1 pathway are resistant to Dox treatment and that constitutive expression of this pathway is predictive for response to adjuvant chemotherapy in patients with breast cancer [18], [25]. We hypothesized that the resistance of *STAT1^H^* tumor clones to Dox is linked to an impaired apoptotic response similar to our recent data for nu61 and SCC61 cell lines [18], [44]. To test this, we chose six cell lines, three of which were *STAT1^H^* (P3M2A, P2M3C and P3M5A; **Figure 3A**) and three cell lines that were *STAT1^L^* (P2M3, P2M6B and P2M5B; **Figure 3A**). Cells were treated with Dox for 24 hours and the apoptotic response was measured by activation of caspases 3/7 (see Methods). Dox activated caspases 3/7 in all the tested cell lines, but the three *STAT1^L^* cell lines had a significantly higher apoptotic response compared to *STAT1^H^* cell lines (**Figure 7**). The mean level of apoptosis induction in *STAT1^L^* cells was 34.1±5.7 fold, while in *STAT1^H^* cells it was 18.3±1.9 fold relative to untreated cells (Student's two-tailed t-test, p = 0.013). It is noteworthy that the two *STAT1^L^* cell lines with the maximal apoptotic response (P2M6B and P2M5B) were also sensitive to IFNγ, while the two *STAT1^H^* cell lines with the minimal apoptotic response (P3M2A and P2M3C) were resistant to IFNγ (**Figures 3A** **,** **5** **and** **Table S2**). P3M5A and P2M3 had intermediate levels of Dox-induced apoptosis and were characterized by intermediate levels of resistance to IFNγ (**Figure 5** **and** **Table S2**). These results support two conclusions. First, the STAT1-dependent development of tumor clones with an increased propensity for lung colonization and resistance to IFNγ is associated with the development of resistance to Dox. Second, this resistance is linked to impaired apoptotic response through suppression of caspase 3/7 activation, which is consistent with our previous reports [18], [44].

### Development of tumor clones proficient in lung colonization and resistant to genotoxic stress is mechanistically connected to *STAT1*

Previously, we showed that stable knockdown of *STAT1* in IR-resistant and IFNα/IFNγ-resistant tumor cells led to radiosensitization [18], suggesting that STAT1 is one important mediator in the IR-resistant tumor phenotype. We used the same approach to test the hypothesis that STAT1 mediates the development of an aggressive pro-lung colonization phenotype as described here. We selected the cell line with the highest *STAT1* score from the P2 STAT1^H^ panel (P2M5A) (**Figure 3A** **and** **Table S1**). Stable *STAT1* knockdowns of this cell line were generated using shRNA (see Methods). For *in vivo* colonization experiments, we tested P2M5A-24 and P2M5A-26 cell lines as they had maximal suppression of *STAT1* with 6- and 12-fold suppression, respectively, as measured by QRT-PCR (results not shown).

**Figure 8:**
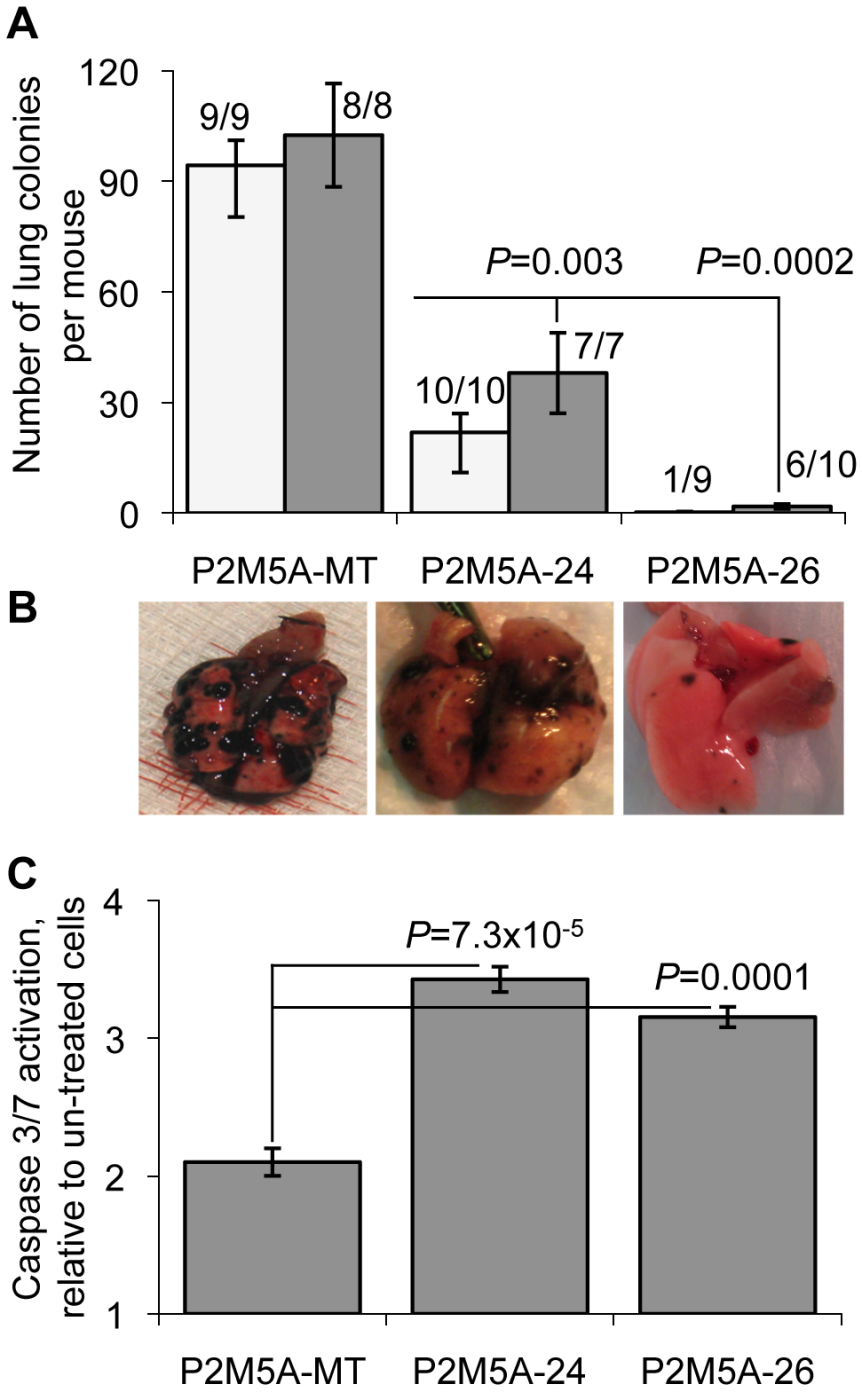
Knockdown of STAT1 using shRNA mitigates aggressive phenotype and restores sensitivity to doxorubicin. (A) Number of lung colonies per mouse produced by control-transfected (P2M5A-MT) and shRNA knockdown (P2M5A-24 and P2M5A-26) cell lines. Shown are data for 2 (white bars) and 3 (gray bars) weeks after IV injection (mean±SEM). (B) Representative images of lungs, colonized by respective cell lines. (C) Caspase 3/7 activation as a measure of apoptosis in control (P2M5A-MT) and shRNA knockdown (P2M5A-24 and P2M5A-26) cells exposed to doxorubicin (1 µM) for 24 hours.

Lungs were explanted and colonies counted at two and three weeks after IV injection. P2M5A cells transfected with the control vector produced lung metastases in 100% of mice at two and three weeks with the mean number of lung metastases per mouse 94.3±6.5 at two weeks and 102±39.6 at three weeks (**Figure 8A**). Six-fold suppression of *STAT1* with shRNA 24 led to a 4–5 fold suppression in lung colonization (Student's two-tailed t-test; p = 0.003), although 100% of mice still developed lung metastases at two and three weeks. However, 12-fold suppression of *STAT1* with shRNA 26 corresponded to a 56-fold suppression (Student's two-tailed t-test; p = 0.0002) in lung colonization with a decrease in the incidence of lung metastases from 100% to 11% at two weeks (Fisher's exact test; p = 1.7×10^−5^) and 60% to 100% at three weeks (Fisher's exact test; p = 0.018; **Figure 8A-B**). These data demonstrate that *STAT1* signaling is a key factor controlling the propensity for B16F1 melanoma cells to colonize the lung and that suppression of *STAT1* can convert the tumor phenotype to poor lung colonizers.

Considering our previous observations of a relationship between expression of the IFN/STAT1 pathway, colonization propensity, and resistance to genotoxic stress, we investigated the effects of *STAT1* knockdown in P2M5A (IFN/STAT1 pathway overexpressing) cells on the response to treatment with Dox. Knockdown of *STAT1* by both shRNA constructs (24 and 26) led to a significant activation of apoptotic response following treatment with Dox compared with the control-vector transfected cell line as measured by activation of caspases 3 and 7 (Student's two-tailed t-test; p = 7.3×10^-5^; **Figure 8C**). These results are consistent with our previous finding that *STAT1* knockdown causes tumor sensitization to genotoxic stress [18]. Taken together these results suggest that *STAT1* plays an important role in the lung colonization ability of B16F1 clones and derivatives and that *STAT1* provides a molecular link between colonization propensity and resistance of metastatic tumor clones to cytotoxic stimuli.

## Discussion

The relationship between overexpression of the IFN/STAT1 pathway and malignancy was initially described for breast tumors [45], [46]. Subsequently, Chung et al. compared gene expression patterns in lung and breast cancers and found two common functional clusters – a proliferation cluster and an IFN/STAT1 cluster [47]. However, the biological significance of overexpression of the IFN/STAT1 pathway remained unclear. We previously demonstrated an association between an IR-resistant phenotype and overexpression of IFN/STAT1 pathway in head and neck cancer cell lines [17]. Furthermore, stable transfection of *STAT1* in the radiosensitive SCC61 cell line led to the formation of an IR- and IFN-resistant phenotype, thus confirming that constitutive overexpression of STAT1 conferred resistance to genotoxic stress. We then demonstrated that selection of SCC61 against IFNγ and IFNα led to a similar IR- and IFN-resistant phenotype [18].

These observations lead us to hypothesize that the development of tumor clones overexpressing the IFN/STAT1 pathway is associated with selection against IFNs, and perhaps other STAT1-activating ligands, secreted by the host microenvironment leading to the selection of cells resistant to the cytotoxic effects of transient STAT1 activation. This resistance is also associated with resistance to IR and chemotherapy, a characteristic of aggressive tumors [18], [25]. Indeed, in an *ex vivo* co-culture model it was demonstrated that stromal fibroblasts can secrete IFNs, activating the IFN/STAT1 pathway in breast cancer cells, and that this pathway is associated with poor prognosis in patients with breast cancer due to the development of metastases [48]. Our current data are consistent with these observations [25].

Our two most important findings reported here are that we demonstrate using an *in vivo* syngeneic model that the development of tumor clones within the host microenvironment leads to the preferential selection of clones constitutively overexpressing the IFN/STAT1 pathway with an associated improved ability to colonize the lung microenvironment. Second, we confirmed that these clones are resistant to genotoxic insult delivered by cytotoxic cytokines, IR, and chemotherapy. These findings raise questions about the potential role of the IFN/STAT1 pathway in metastasis development.

Metastasis emerges as a result of genetic and epigenetic evolution driven by selection pressures created by the host microenvironment and within the tumor itself. A minority of cells from the heterogeneous tumor cell population survive these selection pressures (see [49] for review). The majority of tumor cells are eliminated during the metastatic cascade through multiple microenvironmental stresses including tissue barriers, regulation of nutrients and energy supplies through angiogenesis and hypoxia, interactions with the extracellular matrix, shear stresses within the bloodstream, networks of the cytotoxic cytokines, and tumor surveillance by the immune system (see [49]–[51] for reviews). As a result, only tumor cells with intrinsic properties that provide advantages in this multi-step selection process will survive and colonize distant sites. In this regard, we asked whether overexpression of the IFN/STAT1 pathway provides such a selective advantage to tumor cells, thereby leading to improved lung colonization.

To investigate these questions we used a syngeneic B16 mouse melanoma lung colonization model [26]–[28] (see [2] for review). Given the above mentioned data regarding the complexity and multi-stage nature of the metastatic process, we used the total number of surface lung colonies formed by the parental B16F1 and its derivatives as the endpoint to measure metastatic propensity. The individual lung colonies, which are believed to be the progeny of a single lung colonizing tumor cell [52], were used to establish cell lines in which we examined levels of expression of the IFN/STAT1 pathway. Also, given that IFN/STAT1 signaling involves hundreds of genes [31], [32], we used an expressional score calculated from the expression of five marker genes (see **Figure 1D** and Methods). This score allowed us to designate cell lines as high expressors (*STAT1^H^*) and low expressors (*STAT1^L^*) of the IFN/STAT1 pathway. The combination of these approaches allowed us to screen a panel of 55 cell lines representing three sequential passages of B16F1 through C57BL/6 mice (**Table S1**). We were then able to identify relationships between expression levels of the IFN/STAT1 pathway, resistance of tumor clones to cytotoxic agents, and the ability of tumor clones to colonize lungs.

A striking observation was that after only the first passage (P1), established cell lines obtained from the lung colonies clearly segregated into the *STAT1^H^* and *STAT1^L^* genotypes (**Figure 1D**). Furthermore, *STAT1^H^* clones demonstrated a 10-fold increase in their ability to colonize the lungs compared to *STAT1^L^* clones (**Figure 2**). These results were further confirmed by propagation of *STAT1^H^* clones in the P2 and P3 *in vivo* passages demonstrating both increased frequency of *STAT1^H^* clones and an increase in the level of IFN/STAT1 pathway up-regulation. In parallel, in each passage the ratio of *STAT1^H^* to *STAT1^L^* clones increased. In the P1 passage we observed only 2.1±1.2 colonies per mouse, while in the P3 passage we observed an almost 20-fold increase in lung colonization (37.9±18.7 colonies per mouse; **Figures 1** **and** **3**). These results demonstrate that tumor clones with increased expression of the IFN/STAT1 pathway possess an aggressive phenotype with an enhanced ability to colonize the lungs.

Previously, Fidler and Kripke demonstrated that some monoclones of B16F1 have preexistent abilities to aggressively colonize the lungs [39]. We used a similar approach but focused on expression of the IFN/STAT1 pathway (**Figure 4A**). We concluded that the population of B16F1 cells contains preexisting *STAT1^H^* variants prior to *in vivo* passage. According to our prior and current observations, these cells are resistant to cytotoxic and genotoxic stress, are selected by the lung microenvironment, and are more proficient at colonizing the lungs. Our results suggest that a *STAT1^H^* status is at least in part responsible for the preexistent ability of B16F1 cells to colonize the lungs. Additionally, we propose that significant up-regulation of the IFN/STAT1 pathway *in vivo* likely creates a negative selection pressure against *STAT1^L^* clones that are not resistant to the cytotoxic effects of this pathway, thereby selecting for *STAT1^H^* clones (**Figure 4B**). Importantly, our co-culture data suggest that the lung microenvironment likely plays a significant role in this selection (see [49], [51], [53]).

We tested the resistance of *STAT1^H^* and *STAT1^L^* cell lines and tumors to IFNγ, IR, and Dox (**Figures 5** **–** [Fig pone-0005821-g006] **7**). *STAT1^H^* clones were resistant to all treatments while *STAT1^L^* clones were sensitive. This is consistent with our previous data obtained with human head and neck cell lines SCC61 and nu61 [17], [18], [25], [44]. Fryknas et al [22] showed that overexpression of the IFN/STAT1 pathway in myeloma cell lines is associated with cross-resistance to IR and Dox. Recent observations from our laboratory indicate that resistance to STAT1 is also associated with resistance to the death ligands of the TNF receptor superfamily that are also involved in the control of tumor development [44]. Therefore, it is reasonable to propose that once tumor cells of different types acquire resistance to the IFN/STAT1 pathway and are then selected based on this resistance, they are also intrinsically resistant to a wide variety of cytotoxic and genotoxic insults.

Finally, to provide mechanistic evidence of the dependence of *STAT1* on the observed aggressive *STAT1^H^* genotype, we generated stable *STAT1* knockdowns in the P2M5A cell line, which was the highest overexpressor of *STAT1^H^* in the P2 passage (**Figure 3**). *STAT1* knockdown completely reversed the tumor phenotype, significantly reducing lung colonization while significantly increasing sensitivity to Dox (**Figure 8**). These results are consistent with our previous report, in which stable knockdown of *STAT1* in nu61 (*STAT1^H^* genotype) led to radiosensitization both *in vitro* and *in vivo* [18]. Fryknas et al [22] reported similar effects when inhibiting *STAT1* in myeloma cell lines by fludarabine with sensitization to both IR and Dox. Most recently Hui et al [24] found overexpression of *STAT1* in renal cell carcinoma relative to normal kidney cells and were subsequently able to radiosensitize tumor cells *in vitro* by suppressing *STAT1* using siRNA or fludarabine. Taken together with our current results these data suggest that *STAT1* is tightly associated with resistance to genotoxic stress and provides a promising target for radio-and chemo-sensitization of primary tumors and perhaps distant metastasis.

The observation that constitutive overexpression of the IFN/STAT1 pathway confers aggressive and therapy resistant tumor clones leaves several unanswered questions which need to be explored including the mechanisms by which activation of the IFN/STAT1 pathway switches the pathway from “cytotoxic” to “pro-survival” and the mechanisms that underlie this switch. Recently, we reported that constitutive overexpression of the STAT1 pathway is associated with suppression of caspases 8, 9, and 3 and therefore with suppression of the apoptotic response in radiation and interferon resistant tumor cells [44]. In the current report we also demonstrated that *STAT1^H^* clones have an impaired apoptotic response compared to *STAT1^L^* clones (**Figure 8**). Taken together these results suggest that resistance to the IFN/STAT1 pathway is associated with impaired apoptosis. Our findings suggest that once cells become resistant to apoptosis they can survive activation of the pro-apoptotic branch of the IFN/STAT1 pathway [54]. Still, we cannot exclude that a mechanisms other than apoptosis may also lead to IFN/STAT1 pathway resistance [55]–[57].

In a previous study we also suggested that due to the complexity of the network of STAT1-dependent genes, suppression of the cytotoxic branch of IFN/STAT1 pathway [44] may reveal STAT1-dependent genes with radioprotective and pro-survival functions [18]. Indeed, STAT1 has been reported to control pro-survival genes such as *MCL1* [58], *IFITM1* or *Leu-13* [59], multidrug resistance vault proteins [60], and *USP18* (*UBP43*), which mediate protection from IFN-induced cytotoxicity [61]. Importantly, we previously reported that *MCL1, IFITM1,* and *USP18* are co-expressed with *STAT1* in the IR- and IFN-resistant nu61 tumor [17]. We believe that systematic profiling of *STAT1* knockdowns combined with biological validation will reveal the essential and potentially oncogenic genes controlled by STAT1.

Overall our current data demonstrate that B16F1 syngeneic model of lung metastasis supports our hypothesis that the overexpression of the IFN/STAT1 pathway is an important mediator in the development of an aggressive lung colonizing phenotype that is resistant to negative selection pressures from the lung microenvironment and concurrently resistant to conventional cancer therapy including IR and chemotherapy. Future experiments are needed to elucidate the mechanistic functions of STAT1 that confer this aggressive and therapy-resistant phenotype. Recent reports and our current observations strongly suggest that STAT1 provides a promising target for sensitization of primary tumors and perhaps distant metastasis to anti-tumor therapy.

## Methods

### Cell culture

B16F1 and B16F10 murine melanoma cell lines were obtained from the American Type Culture Collection (Manassas, VA) and were cultured in RPMI 1640 media (Invitrogen, Carlsbad, CA) supplemented with 10% fetal bovine serum (Atlanta Biologicals, Lawrenceville, GA), 100 U/mL penicillin, and 100 mg/mL streptomycin (Invitrogen, Carlsbad, CA). Parental B16F1 cell lines and daughter cell lines, obtained after *in vivo* passages, were maintained in culture with the same media with 5% CO_2_ at 37°C or stored in liquid nitrogen. For generation of STAT1 knockdowns we used commercial shRNA constructs (Sigma, Saint Louis, MI), as described elsewhere [18]. Cells were cultured the same as parental B16F1 with the addition of 1 mg/ml of puromycin.

### Mice

C57BL/6-Ncr mice were obtained from FCRI-Taconic (Germantown, NY) and B6.129S7-IFNγ^tm1Ts^−/− (IFNγ-null phenotype) were obtained from Jackson Laboratories (Bar Harbour, ME). Animals were 5–7 weeks of age at the beginning of all experiments. The care and treatment of experimental animals was in accordance with institutional guidelines at the University of Chicago.

### Tumor cell injections

C57BL/6 mice were injected i.v. via the internal jugular (IJ) vein or tail vein with tumor cells. For the IJ injections mice were anesthetized with a Ketamine mixture. After shaving the neck/clavicular area, the skin was incised and vein dissected free. 100 mL of cellular suspension was delivered under direct visualization and without spillage. Cyanoacrilate was used for skin closure. 5×10^4^ tumor cells were injected i.v. per mouse.

### Selection of cell lines from IFN/STAT1-expressing tumor colonies

We followed the model of experiments from Fidler and colleagues [26] with the addition of screening established cell lines for expression of IFN/STAT1 pathway genes. Parental B16F1 cell lines were injected in C57BL/6 mice and lung colonies were harvested using gross dissection techniques. Each colony was divided with one half used for QRT-PCR profiling and the other half used for establishment of the primary cell lines (P1 panel). QRT-PCR profiling of the P1 panel IFN/STAT1 pathway is described in Methods Section 6. *STAT1^H^* (high expressors) and *STAT1^L^* (low expressors) P1 cell lines were separately pooled and injected into the next group of mice to generate the P2 panel of cell lines. To represent progeny of P1 *STAT1^H^* and P1 *STAT1^L^* cell lines the P2 cell lines were designated as P2^H^ and P2^L^, respectively. Finally, P2^H^ cell lines were pooled and passed *in vivo* as described above to generate a P3^H^ panel of cell lines (See **Figure S2** for a schematic representation).

### Collection of cell lines and in vivo samples

One-half of each lung colony was placed into an Eppendorf tube, snap-frozen in liquid nitrogen, and transferred to a −80°C freezer. The other half of each sample was used to establish an independent cell line. To accomplish this the lung colony fragment was placed into a single well of a 24-well plate, covered by 1 ml of culture media (see Methods section 1), and incubated in 5% CO_2_ at 37°C until a monolayer of cells, morphologically similar to B16F1, was apparent under microscopic observation. The lung colony fragment was removed, the cells were detached by trypsinization, and then the cells were transferred to parallel 24-well plates and cultured for 2-4 days. Cell cultures that produced a consistent confluent monolayer were collected, frozen, and used in the subsequent experiments. All established cell lines were stored in liquid nitrogen.

### IFN/STAT1 pathway scoring system based on QRT-PCR profiling

To quantify IFN/STAT1 pathway expression levels in multiple samples using QRT-PCR, we used a five gene marker set (STAT1, IFIT1, IFIT3, IFITM1, and MX1) (see [18]). Primers for the mouse marker genes are presented in **Table S3**. The QRT-PCR technique used in these experiments is previously described [62]. For calculation of the IFN/STAT1 pathway expression score (ES) we used the equation 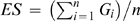, where *G_i_* = *log_2_R*, *R* = fold ratio relative to B16F1, and *n* = number of genes.

### Resistance to IFNγ

Cell lines were plated in 96–well plates (5×10^3^ cells/well) and treated with 10 ng/ml of murine IFNγ (R&D Systems, Minneapolis, MN). 48 hours later cell survival was determined using the Promega CellTiter 96* Nonradioactive Cell Proliferation Assay (MTT) (Promega, Madison, WI) according to the manufacturer's instructions. Viability was calculated relative to a B16F1 control.

### Resistance to ionizing radiation

*In vivo* radioresistance of *STAT1^H^* and *STAT1^L^* cell lines was determined as previously described [42]. Briefly, B16F1, P2M3C (*STAT1^H^*), and P2M6B (*STAT1^L^*) were injected subcutaneously (1×10^7^ cells in 100 ml PBS) into the right hind limb of C57BL/6 mice. Five to seven days after injection the tumor volume was 150–200 mm^3^. At the initiation of treatment (day 0) animals were divided into appropriate treatment groups and randomized with respect to the tumor size. A single dose of 20 Gy was delivered using a Phillips orthovoltage X-ray generator operating at 250 kV 15 mA. Tumor volume was determined by direct measurement with calipers and calculated by the formula (length×width×depth/2 [42]. At the conclusion of each experiment animals were euthanized using CO_2_ followed by cervical dislocation in accordance with the institutional guidelines.

### Sensitivity to Doxorubicin and apoptosis measurement

To test the effects of IFN/STAT1 pathway expression levels on the apoptotic response to Dox we used three *STAT1^H^* cell lines (P3m2a, P2m3c, P3m5a) and three *STAT1^L^* cell lines (P2m3, P2m6b, and P2m5b). We selected one *STAT1^H^* cell line (P2m5a) and generated *STAT1* knockdowns (see above) that were also tested for Dox sensitivity. Cells were plated at a density of 5×10^3^ cells per well in 96-well plates in triplicates for 24 hours and then treated with 1.0 µM Dox for 24 hours. Apoptosis was measured using the Promega Caspase-Glo 3/7 assay (Madison, WI). Luminescence was measured 30 minutes after addition of the Caspase-Glo reagent using a TD-20/20 Luminometer. Signal intensities for treated samples were background (no cells) subtracted and divided by the intensity of an untreated control to calculate relative induction of apoptosis.

### Co-culture of B16F1 with C57BL/6 lung tissue

B16F1 cells were plated in triplicate at a density of 1×10^6^ per well in a 6-well plate and incubated for 24 hours. 0.4 µm polyester membrane transwell inserts (Costar 3450, Corning, NY) were placed on top of each well and 1×10^6^ B16F1 cells (mono-culture) or lung tissue removed from C57BL/6 mice that was rinsed with warm PBS (co-culture) were added. B16F1 cells on the bottom of the wells were removed after 8 and 24 hours of mono- or co-cultured incubation. Expression of the IFN/STAT1 pathway was measured relative to B16F1 monoculture as described above.

### Detection of pre-existing *STAT1^H^* clones

B16F1 cells were plated at 1 cell/well (monoclones) in a 96-well plate (Nunclon Surface, Denmark). After one week of growth, colonies were transferred to 24-well plates and grown to confluence. 20 monoclone cultures were collected using Trizol reagent and analyzed using QRT-PCR. Scoring and quantitative estimation of the data are described above.

## Supporting Information

Figure S1 Correlation between gene array expression levels and QRT-PCR expression levels. Marker genes used for IFN/STAT1 expression score are labeled. Dashed line  =  95% confidence interval.
(0.19 MB PPT)

Figure S2 Schematic representation of B16 P1, P2H, P2L, and P3H passages. Note that steps within circle were repeated for each passage to characterize lung colonies using QRT-PCR for IFN/STAT1 pathway expression scores and to develop stable cell lines in vitro.
(0.20 MB PPT)

Table S1 STAT1 individual marker gene QRT-PCR levels and overall IFN/STAT1 pathway expression score for all cell lines relative to B16F1.
(0.12 MB DOC)

Table S2 IFNÎ^3^ resistance and STAT1 expression score for cell lines tested.
(0.03 MB DOC)

Table S3 IFN/STAT1 marker gene QRT-PCR primers
(0.03 MB DOC)
