# Supplementary figures and images for: STAT1 Pathway Mediates Amplification of Metastatic Potential and Resistance to Therapy
Source: PLoS One. 2009 Jun 8;4(6):e5821. doi: 10.1371/journal.pone.0005821 (PMC2688034; doi:10.1371/journal.pone.0005821)

## Slide 1
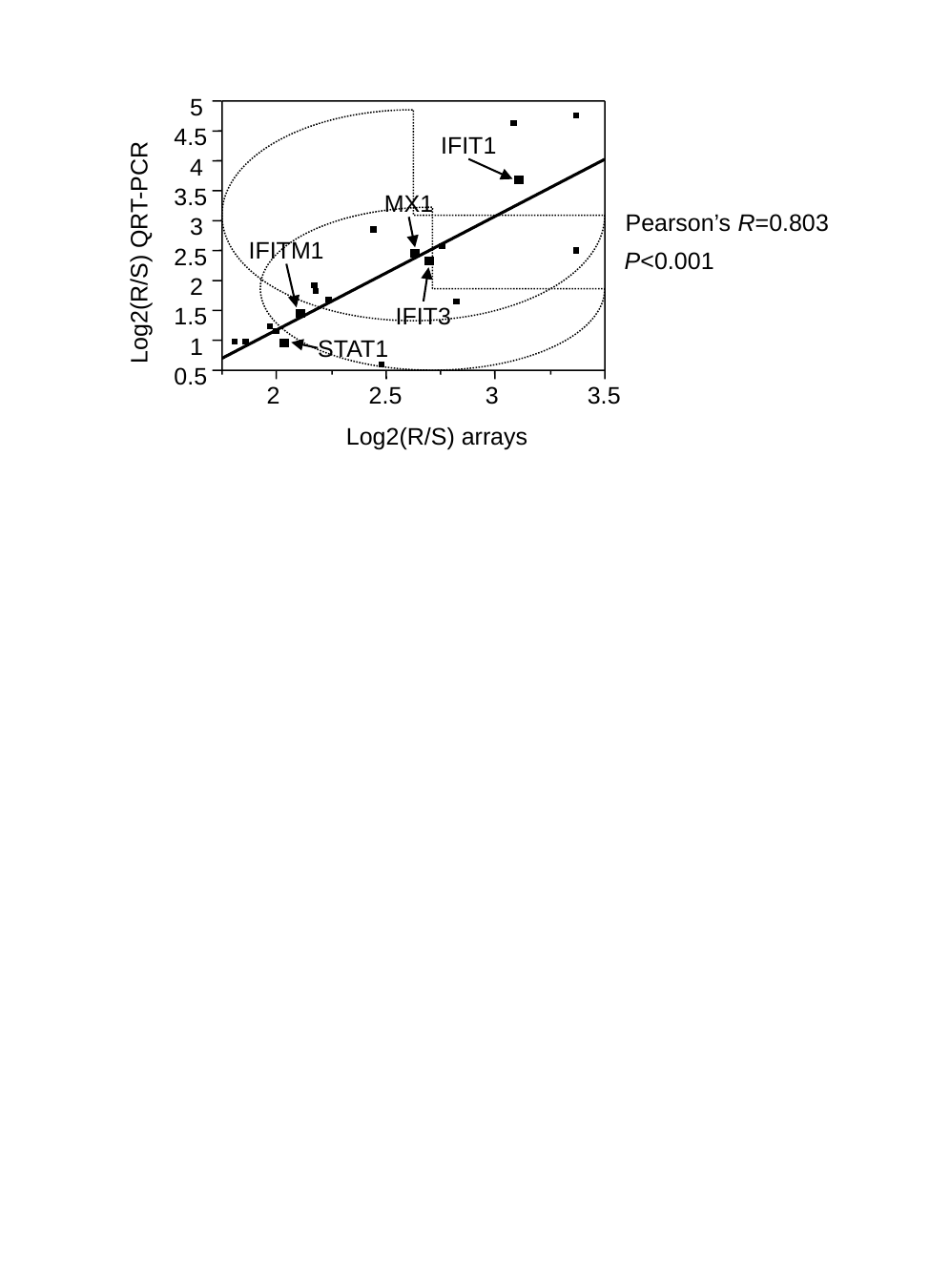

5
4.5
IFIT1
IFIT1
4
3.5
MX1
MX1
Pearson’s R=0.803
3
IFITM1
Log2(R/S) QRT-PCR
2.5
P<0.001
IFITM1
2
IFIT3
1.5
IFIT3
1
STAT1
STAT1
0.5
2
2.5
3
3.5
Log2(R/S) arrays

Supplement: Figure S1 — Correlation between gene array expression levels and QRT-PCR expression levels. Marker genes used for IFN/STAT1 expression score are labeled. Dashed line = 95% confidence interval. (0.19 MB PPT) [file pone.0005821.s001.ppt]
