# Supplementary material for: STAT1 Pathway Mediates Amplification of Metastatic Potential and Resistance to Therapy
Source: PLoS One. 2009 Jun 8;4(6):e5821. doi: 10.1371/journal.pone.0005821 (PMC2688034; doi:10.1371/journal.pone.0005821)
Supplement: Figure S2 — Schematic representation of B16 P1, P2H, P2L, and P3H passages. Note that steps within circle were repeated for each passage to characterize lung colonies using QRT-PCR for IFN/STAT1 pathway expression scores and to develop stable cell lines in vitro. (0.20 MB PPT) [file pone.0005821.s002.ppt]

## Slide 1
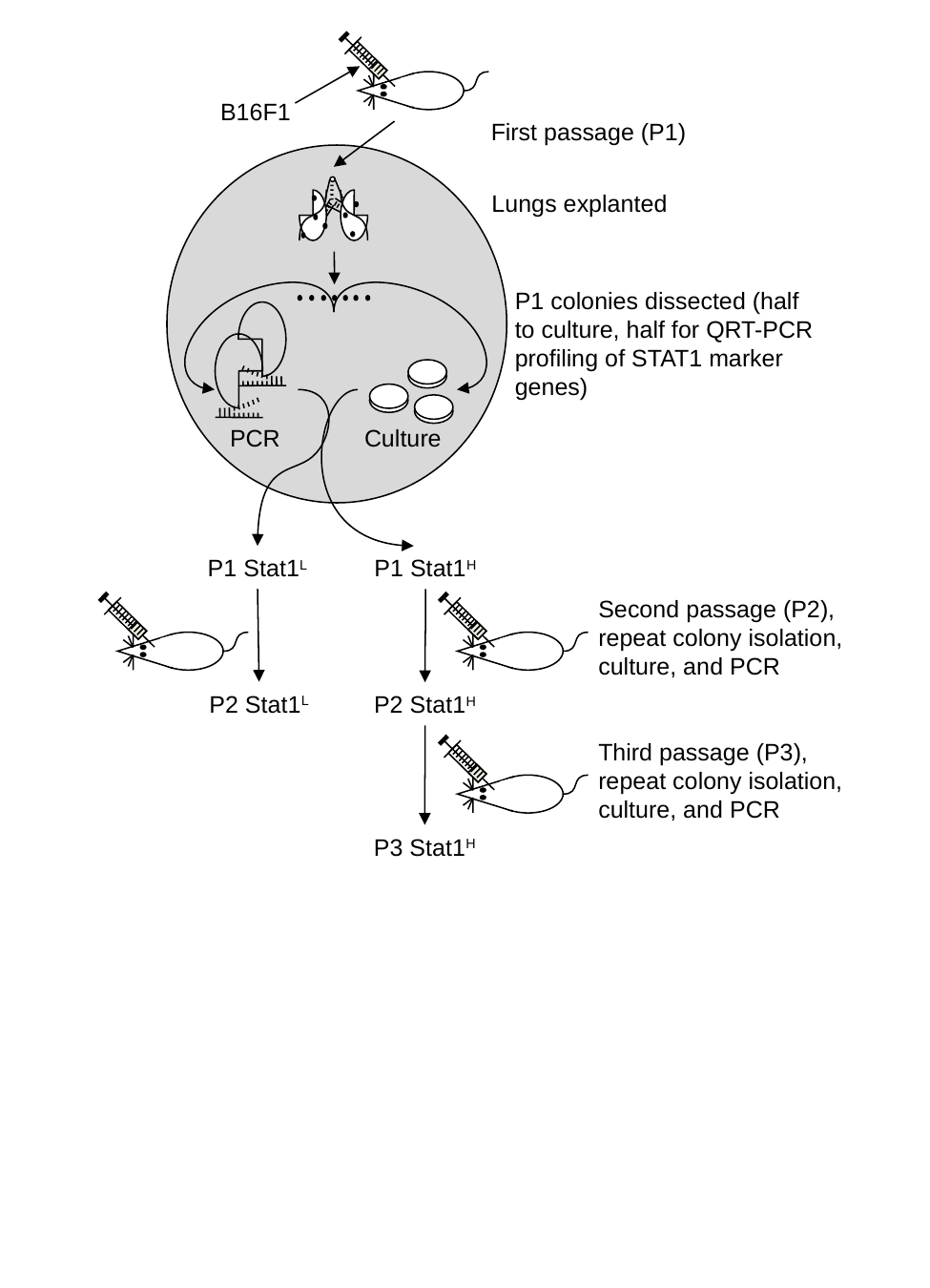

B16F1
First passage (P1)
Lungs explanted
P1 colonies dissected (half to culture, half for QRT-PCR profiling of STAT1 marker genes)
Culture
PCR
P1 Stat1L
P1 Stat1H
Second passage (P2), repeat colony isolation, culture, and PCR
P2 Stat1L
P2 Stat1H
Third passage (P3), repeat colony isolation, culture, and PCR
P3 Stat1H
