# Supplementary material for: STAT1 Pathway Mediates Amplification of Metastatic Potential and Resistance to Therapy
Source: PLoS One. 2009 Jun 8;4(6):e5821. doi: 10.1371/journal.pone.0005821 (PMC2688034; doi:10.1371/journal.pone.0005821)
Supplement: Table S1 — STAT1 individual marker gene QRT-PCR levels and overall IFN/STAT1 pathway expression score for all cell lines relative to B16F1. (0.12 MB DOC) [file pone.0005821.s003.doc]

**Supplementary Table 1**

STAT1 individual marker gene QRT-PCR levels and overall IFN/STAT1 pathway expression score for all cell lines relative to B16F1.

| **Sample** | **Group** | **STAT1** | **MX1** | **IFITM1** | **IFIT3** | **IFIT1** | **Expression Score** | **IFN/STAT1 pathway designation** |
| --- | --- | --- | --- | --- | --- | --- | --- | --- |
| P1M1a | P1L | -0.64 | -0.41 | -1.76 | -1.02 | -0.66 | -0.90 | STAT1^L^ |
| P1M1d | P1H | 0.91 | 0.76 | 0.56 | 0.85 | 0.99 | 0.82 | STAT1^H^ |
| P1M3e | P1H | 0.41 | 1.55 | 1.21 | 1.66 | 1.22 | 1.21 | STAT1^H^ |
| P1M4d | P1L | -0.18 | -0.11 | -0.49 | 0.06 | 0.08 | -0.13 | STAT1^L^ |
| P1M5c | P1L | -0.41 | -0.06 | -0.17 | -0.43 | -0.36 | -0.28 | STAT1^L^ |
| P1M5b | P1L | 0.08 | -0.42 | -0.72 | -0.53 | -0.09 | -0.33 | STAT1^L^ |
| P1M7 | P1H | 1.16 | 0.05 | 0.71 | 1.45 | 1.07 | 0.89 | STAT1^H^ |
| P1M8d | P1L | -0.09 | 0.11 | 0.15 | -0.06 | -0.81 | -0.14 | STAT1^L^ |
| P2M1C | P2H | 0.69 | 1.59 | 1.01 | 1.00 | 0.37 | 0.94 | STAT1^H^ |
| P2M2B | P2H | 0.21 | 0.95 | 1.23 | 0.47 | 0.12 | 0.60 | STAT1^L^ |
| P2M3B | P2H | 0.64 | 0.46 | 0.22 | -0.13 | -0.43 | 0.15 | STAT1^L^ |
| P2M3C | P2H | 0.68 | 3.84 | 2.98 | 2.65 | 1.62 | 2.36 | STAT1^H^ |
| P2M4B | P2H | 0.62 | -0.38 | 0.15 | -0.51 | -0.48 | -0.12 | STAT1^L^ |
| P2M5A | P2H | 0.04 | 4.47 | 3.70 | 3.60 | 2.52 | 2.87 | STAT1^H^ |
| P2M6 | P2H | 0.79 | 1.55 | 0.87 | 1.38 | 0.44 | 1.00 | STAT1^H^ |
| P2M7A | P2H | 0.89 | 2.06 | 1.31 | 1.28 | 0.29 | 1.17 | STAT1^H^ |
| P2M7B | P2H | 0.55 | 1.31 | 1.58 | 0.37 | -0.32 | 0.70 | STAT1^L^ |
| P2M9 | P2H | 1.33 | 1.57 | 1.44 | 1.20 | 0.31 | 1.17 | STAT1^H^ |
| P2M10 | P2H | 0.93 | 0.14 | -0.02 | 0.06 | -0.29 | 0.16 | STAT1^L^ |
| P2M12 | P2H | 1.20 | -0.91 | -0.37 | -0.09 | -0.48 | -0.13 | STAT1^L^ |
| P2M1B | P2H | 1.23 | -0.72 | -1.19 | 0.23 | 0.09 | -0.07 | STAT1^L^ |
| P2M1A | P2H | 1.08 | -0.10 | -1.50 | 0.52 | 1.30 | 0.26 | STAT1^L^ |
| P2M3A | P2H | 1.89 | 2.45 | 0.91 | 1.80 | 1.25 | 1.66 | STAT1^H^ |
| P2M4A | P2H | 0.53 | -1.05 | -1.42 | -0.80 | -0.68 | -0.69 | STAT1^L^ |
| P2M5B | P2H | 1.09 | 1.19 | 0.51 | 0.58 | 0.51 | 0.78 | STAT1^L^ |
| P2M8A | P2H | 0.37 | -0.44 | -0.75 | -0.53 | -0.61 | -0.39 | STAT1^L^ |
| P2M8B | P2H | 1.03 | 0.12 | -0.73 | 0.29 | -0.16 | 0.11 | STAT1^L^ |
| P2M11A | P2H | 0.70 | 1.35 | 0.52 | 0.90 | 0.94 | 0.88 | STAT1^H^ |
| P2M2A | P2H | 1.90 | 3.33 | 2.25 | 2.22 | 1.48 | 2.24 | STAT1^H^ |
| P2M11B | P2H | 1.09 | 1.47 | 0.60 | 1.06 | 0.90 | 1.02 | STAT1^H^ |
| P2M13A | P2H | 1.11 | 2.53 | 1.98 | 1.42 | 0.79 | 1.57 | STAT1^H^ |
| P2M1B | P2H | -0.80 | -1.19 | -2.77 | -1.41 | -1.51 | -1.54 | STAT1^L^ |
| P2M13C | P2H | 0.34 | 0.70 | -0.22 | 0.51 | 0.24 | 0.31 | STAT1^L^ |
| P2-M1A C | P2L | 1.10 | 1.45 | -0.02 | 1.59 | -0.46 | 0.73 | STAT1^L^ |
| P2-M1B C | P2L | 1.48 | 0.43 | -2.20 | 1.57 | -0.27 | 0.20 | STAT1^L^ |
| P2-M2A C | P2L | 1.15 | 0.77 | -1.42 | 1.48 | -0.19 | 0.36 | STAT1^L^ |
| P2-M3 C | P2L | 0.60 | -1.03 | -4.54 | 0.34 | -1.93 | -1.31 | STAT1^L^ |
| P2-M4 C | P2L | 0.44 | 5.58 | 3.78 | 5.43 | 3.44 | 3.73 | STAT1^H^ |
| P2-M5B C | P2L | 1.83 | -0.43 | -2.59 | 1.03 | -0.58 | -0.15 | STAT1^L^ |
| P2-M6A C | P2L | 1.43 | 1.34 | -0.62 | 2.06 | 0.68 | 0.98 | STAT1^H^ |
| P2-M6B C | P2L | 1.17 | -0.19 | -2.21 | 0.73 | -0.95 | -0.29 | STAT1^L^ |
| P2-M7 C | P2L | 1.56 | 0.56 | -1.96 | 1.21 | -1.08 | 0.06 | STAT1^L^ |
| P3M1A | P3H | 2.35 | -1.27 | -3.50 | -0.18 | 0.11 | -0.50 | STAT1^L^ |
| P3M1B | P3H | 2.51 | -1.74 | -3.67 | 0.07 | 0.24 | -0.52 | STAT1^L^ |
| P3M2A | P3H | 4.80 | 0.42 | -2.64 | 3.92 | 4.68 | 2.24 | STAT1^H^ |
| P3M2B | P3H | 2.65 | -0.47 | -3.83 | 2.24 | 2.23 | 0.57 | STAT1^L^ |
| P3M3A | P3H | 3.13 | 0.64 | -3.75 | 3.45 | 3.16 | 1.33 | STAT1^H^ |
| P3M4A | P3H | 4.01 | 1.05 | -2.51 | 3.28 | 3.16 | 1.80 | STAT1^H^ |
| P3M4B | P3H | 3.42 | 2.98 | 2.44 | 3.57 | 2.89 | 3.06 | STAT1^H^ |
| P3M5A | P3H | 0.44 | 4.59 | 3.62 | 3.97 | 3.02 | 3.13 | STAT1^H^ |
| P3M6A | P3H | 1.70 | 3.98 | 3.39 | 3.25 | 2.25 | 2.92 | STAT1^H^ |
| P3M7B | P3H | 3.50 | 1.20 | -1.92 | 3.37 | 2.53 | 1.74 | STAT1^H^ |
| P3M8A | P3H | 3.91 | 1.21 | -3.88 | 4.01 | 3.52 | 1.75 | STAT1^H^ |
| P3M8B | P3H | 2.97 | 2.97 | 2.54 | 2.49 | 2.23 | 2.64 | STAT1^H^ |
| P3M9 | P3H | 2.06 | -0.82 | -4.42 | 0.38 | 0.97 | -0.37 | STAT1^L^ |
