# Supplementary material for: STAT1 Pathway Mediates Amplification of Metastatic Potential and Resistance to Therapy
Source: PLoS One. 2009 Jun 8;4(6):e5821. doi: 10.1371/journal.pone.0005821 (PMC2688034; doi:10.1371/journal.pone.0005821)
Supplement: Table S2 — IFNÎ3 resistance and STAT1 expression score for cell lines tested. (0.03 MB DOC) [file pone.0005821.s004.doc]

**Supplementary Table 2**

**IFNγ resistance and STAT1 expression score for cell lines tested.**

**Sample IFNγ resistance Expression score IFN/STAT1 pathway designation**

P2+M3c 21.16 2.01 STAT1^H^

P2+M5a 20.67 2.52 STAT1^H^

P2+M2a 20.58 1.89 STAT1^H^

P2+M13a 13.93 1.22 STAT1^H^

P3M6a 11.94 2.57 STAT1^H^

P3M8a 9.86 1.41 STAT1^H^

P3M2a 30.59 1.89 STAT1^H^

P3M5a 3.55 2.79 STAT1^H^

P2M7a 18.63 0.82 STAT1^H^

P2M7b 21.44 0.35 STAT1^L^

P2M6 11.15 0.66 STAT1^L^

P2M9 12.92 0.83 STAT1^H^

P1M1 -0.75 -1.24 STAT1^L^

P1M4c -3.83 -0.47 STAT1^L^

P1M5b 1.50 -0.68 STAT1^L^

P2M6b -6.50 -0.63 STAT1^L^

P2M1b 4.31 -0.14 STAT1^L^

P2M5b -2.23 -0.49 STAT1^L^

P2M7 -5.47 -0.29 STAT1^L^

P2M3 -2.82 -1.66 STAT1^L^

P2M7 1.06 -0.29 STAT1^L^

P2M6b 2.19 -0.63 STAT1^L^

P2M6b 2.19 -0.63 STAT1^L^
