# Supplementary material for: STAT1 Pathway Mediates Amplification of Metastatic Potential and Resistance to Therapy
Source: PLoS One. 2009 Jun 8;4(6):e5821. doi: 10.1371/journal.pone.0005821 (PMC2688034; doi:10.1371/journal.pone.0005821)
Supplement: Table S3 — IFN/STAT1 marker gene QRT-PCR primers (0.03 MB DOC) [file pone.0005821.s005.doc]

**Supplementary Table 3**

**IFN/STAT1 marker gene QRT-PCR primers**

**Gene Forward Primer Reverse Primer**

STAT1 agtcggaggccctaatgct ccataatgcacccatcattcca

MX1 gaccataggggtcttgacaa agacttgctctttctgaaaagcc

IFIT1 ctgagatgtcacttcacatggaa gtgcatccccaatgggttct

IFITM1 gacagccaccacaatcaacat cccaggcagcagaagttcat

IFIT3 agtgaggtcaaccgggaatct tctaggtgctttatgtaggcca

GAPDH aacgaccccttcattgac tccacgacatactcagcac
